# THE RELATION OF AGE TO OUTCOMES IN THE DIABETES LIFESTYLE CHANGE PROGRAM CONDUCTED BY VIRGINIA COOPERATIVE EXTENSION

**DOI:** 10.1093/geroni/igac059.2737

**Published:** 2022-12-20

**Authors:** Azin Pourkhalili, Carlin Rafie, Arash Sarshar, Lynn Margheim

**Affiliations:** Virginia Tech, Blacksburg, Virginia, United States; Virginia Tech/Virginia Cooperative Extension, Blacksburg, Virginia, United States; Virginia Tech, Blacksburg, Virginia, United States; Virginia Tech, Blacksburg, Virginia, United States

## Abstract

The National Diabetes Prevention Program established by the Centers for Disease Control and Prevention promotes the implementation of an evidence-based lifestyle change program (LCP) to prevent or delay the onset of diabetes. The LCP is a 12-month program with 26 lessons covering topics on healthy diets, increasing physical activity, managing stress, and coping with triggers, among others. It includes weekly goal setting, food, and physical activity tracking, and group support. The goals of the program are 5–7% sustained weight loss and 150 minutes of physical activity weekly. Little is known about the real-world effectiveness of the LCP in different age groups, particularly in older adults. The aim of this study was to evaluate the effects of age on LCP outcomes (weight loss, average physical activity, program attendance) conducted by Virginia Cooperative Extension from 2017 - 2022. Among 191 participants enrolled in the LCP, 141 (73%) completed eight or more sessions and 56% were above 60 years of age. Results show there was a significant direct relationship (coefficient=0.05, p=0.001) between age and weight loss percentage. Participants older than 60 had significantly (p=0.03) higher average physical activity (215 min per week) compared to those under 60 years old (161 min per week). In addition, participants above 60 had significantly (p=0.03) higher attendance (22 sessions) compared to those under 60 years of age (19 sessions). These findings suggest that targeting different age groups and intervention delivery methods can improve program outcomes.

